# *Rv3737* is required for* Mycobacterium tuberculosis* growth in vitro and in vivo and correlates with bacterial load and disease severity in human tuberculosis

**DOI:** 10.1186/s12879-021-06967-y

**Published:** 2022-03-14

**Authors:** Qing Li, Zhangli Peng, Xuefeng Fu, Hong Wang, Zhaoliang Zhao, Yu Pang, Ling Chen

**Affiliations:** 1 Tuberculosis Division of Respiratory and Critical Care Medicine, Affiliated Hospital of Zunyi Medical University, No. 149, Dalian Road, Huichuan District, Zunyi City, 563000 Guizhou Province China; 2 Department of Bacteriology and Immunology, Beijing Key Laboratory for Drug Resistant Tuberculosis Research, Beijing Chest Hospital, Capital Medical University, Beijing Tuberculosis and Thoracic Tumor Institute, Area 2, Yard 9, Beiguan Street, Yongzhun Town, Tongzhou District, Beijing, 101100 China

**Keywords:** *Rv3737*, Mycobacterium tuberculosis, Virulence, Transporter

## Abstract

**Background:**

Rv3737 is the sole homologue of multifunctional transporter ThrE in *Mycobacterium tuberculosis* (*Mtb*). In this study, we aimed to investigate whether this transporter participates in vitro and in vivo survival of *Mtb*.

**Methods:**

To characterize the role of *Rv3737*, we constructed and characterized a *Mtb* H37RvΔRv3737. This strain was evaluated for altered growth rate and macrophage survival using a cell model of infection. In addition, the comparative analysis was conducted to determine the association between *Rv3737* mRNA expression and disease severity in active pulmonary TB patients.

**Results:**

The H37RvΔRv3737 strain exhibited significantly slow growth rate compared to H37Rv-WT strain in standard culture medium. Additionally, the survival rate of H37Rv-WT strain in macrophages was 2 folds higher than that of H37RvΔRv3737 at 72 h. A significantly higher level of TNF-α and IL-6 mRNA expression was observed in macrophages infected with H37RvΔRv3737 as compared to H37Rv-WT. Of note, *Rv3737* expression was significantly increased in clinical *Mtb* isolates than H37Rv-WT. The relative expression level of *Rv3737* was positively correlated with lung cavity number of TB patients. Similarly, the higher *Rv3737* mRNA level resulted in lower C(t) value by Xpert MTB/RIF assay, demonstrating that a positive correlation between *Rv3737* expression and bacterial load in TB patients.

**Conclusions:**

Our data takes the lead in demonstrate that the threonine transporter *Rv3737* is required for in vitro growth and survival of bacteria inside macrophages. In addition, the expression level of *Rv3737* may be associated with bacterial load and disease severity in pulmonary tuberculosis patients.

**Supplementary Information:**

The online version contains supplementary material available at 10.1186/s12879-021-06967-y.

## Background

Tuberculosis (TB), caused by *Mtb* complex, constitutes a major global health threat. It estimates that one third of the world’s population is latently infected by the bacterium, and 10.0 million have fallen ill with TB annually [1]. HIV pandemic and the emergence of multidrug-resistant tuberculosis have contributed further to its spread [2, 3]. The life cycle of *Mtb* involves transition stages from infection, dormancy and reactivation, and in certain cases active TB has also been shown to occur as a result of reactivation of latent TB infection [4]. Specially, individuals with immunosuppression have higher odds of TB reactivation compared with normal individuals [5]. Therefore, *Mtb* has evolved many strategies to survive long periods of stress encountered in the human host by reducing its metabolic activity and modulation of the host immune response.

Bacteria are equipped with a broad variety of transport systems [6]. Transport processes play a pivotal role in pathogen metabolism, e.g. for the uptake of nutrients and excretion of harmful agents [7]. Moreover, several amino acid transporters are reported as being associated with pathogenesis [6]. For instance, threonine transporter ThrE is essential for the fitness of *Corynebacterium glutamicum *in vitro growth [8]. Inactivation of *thrE* gene shows reduced growth rate in vitro in medium supplemented with threonine. In addition to threonine, the ThrE carrier serves to export small molecules, indicating that it is a multifunctional transporter that gets rid of metabolic waste products and thus hold more importance to *thrE* in biological fitness [8].

Of note, only two homologues are identified in *Mtb* and *S. coelicolor* [8]. *Rv3737* encodes a 55 Da protein with significant sequence similarity to characterized ThrE protein [9]. As a member of a new translocator family that has never been reported before, it is interesting to investigate whether this multifunctional potential transporter participates in vitro and in vivo survival of *Mtb*. To characterize the role of *Rv3737*, we constructed and characterized an *Mtb* Rv377 deletion mutant (H37RvΔRv3737). This strain was evaluated for altered growth rate and macrophage survival using a cell model of infection. In addition, the comparative analysis was conducted to determine the association between *Rv3737* mRNA expression and disease severity in active pulmonary TB patients.

## Methods

### Bacterial strains, plasmids and cells

The bacterial strains, plasmids and cells used in this study are detailed in Additional file 2: Table S1. *E. coli* DH5α and *E. coli* HB101 cells were grown in Luria-Bertani (LB) broth or LB agar plates at 37 °C. Clinical isolates of *Mtb*, *Mtb* reference strain H37Rv (H37Rv-WT, ATCC27294), *Mycobacterium smegmatis* mc^2^ 155, the Rv3737-overexpressing strain (Msm/pMV261-Rv3737), and empty plasmid pMV261 was electroporated into *M. smegmatis* mc^2^ 155 (Msm/pMV261) were growth on Lowenstein–Jensen (L-J) medium (Encode, Zhuhai, China), Middlebrook 7H9 broth or 7H10 agar plates containing 0.05% Tween 80, 0.5% glycerol and 10% OADC. The selective 7H9 broth or 7H10 agar plate supplemented with 75 µg/ml hygromycin was used to subculture *Mtb Rv3737* knockout strain (H37RvΔRv3737). The bacteria with OD_600_ of 0.6–1.0 were used for in vitro experiments. RAW264.7 cells cultured in DMEM complete medium containing 10% Fetal Bovine Serum.

In addition, 12 clinical *Mtb* isolates were collected from a set of sputum smear-positive and GeneXpert MTB-positive specimens from August 2016 to February 2017. The demographic and clinical characteristics were available from electronic medical records. The number of cavities in the lungs is obtained by reading the images of the patient’s chest CT examination. This study was subject to approval by the Ethics Committees of the Affiliated Hospital of Zunyi Medical University. All patients were≥18 years old and provided written informed consent prior to enrolment.

### Construction of H37RvΔRv3737

**Fig. 1:**
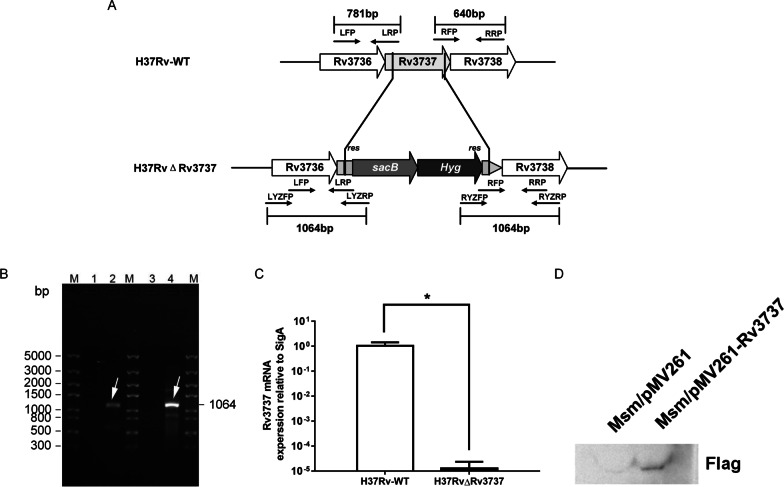
Construction the *Rv3737* knockout *Mtb* strain *and Rv3737 Overexpression Msm strain*.** A** The wildtype *Rv3737* was substituted with DNA fragment encoding hygromycin resistance by homologous recombination.** B** Verification of the *Rv3737* knockout *Mtb* strain by PCR amplification; M: DNA Marker 5000 bps; Lane 2: H37RvΔRv3737 left arm PCR product band; Lane 4: H37RvΔRv3737 right arm PCR product band; H37Rv-WT both arms PCR products have no bands.** C** Verification of the *Rv3737* knockout *Mtb* strain by qPCR, *t* test, *p < 0.01.** D** Verification of the *Rv3737* Overexpression *Msm* strain by Western blotting (full length gels and blots was provided in Additional file 1: Fig. S1 with changes marked)

Using genomic DNA of H37Rv-WT as a template, the *Rv3737* nucleic acid sequence was derived from Genbank of NCBI (https://www.ncbi.nlm.nih.gov/gene/885794). As shown in Fig. 1A, the primers for the upper and lower arms of the *Rv3737* gene and verification primers were designed based on the principle of homologous recombination (Additional file 3: Table S2). Flanking regions comprising upstream and downstream regions of the *Rv3737* gene were amplified by PCR and cloned into the p0004S plasmid containing a hygromycin resistance cassette, the vector was then ligated into the phAE159 plasmid, which was electroporated into *M. smegmatis* mc^2^ 155, and the resulting phage was amplified to obtain a high-titer stock. The high-titer phage was utilized to infect H37Rv-WT, which was plated onto selective 7H10 agar plates. Plates were incubated for 4–8 weeks at 37 °C, which eventually gave rise to the growth of a small number of H37RvΔRv3737 colonies. Colonies were picked, and PCR and qPCR were used to confirm the presence of the hygromycin-gene flanking region and the absence of the *Rv3737* gene (Fig. 1B, C) [10, 11].

### Generation of Msm/pMV261-Rv3737

The full-length sequence of Rv3737 was amplified from H37Rv-WT genomic DNA. Primers for transcriptional level analysis are listed in Additional file 3: Table S2. The fragment was inserted into the downstream of promoter hsp60 within the recombinant pMV261 plasmid. The constructed plasmid was introduced into *M. smegmatis* mc^2^ 155 by electroporation, and the presence of the vector was confirmed by Western Blot. Empty plasmid pMV261 was electroporated into *M. smegmatis* mc^2^ 155, but without Flag tag (Fig. 1D).

### Growth and colonial morphology of Rv3737 knockout strain

H37RvΔRv3737 was inoculated in selective 7H9 broth medium and incubated at 37 °C. Optical density at 600 nm (OD_600_) was detected at intervals of 24 h and the growth curves at 37 °C were obtained. H37RvΔRv3737 was inoculated in selective 7H10 agar plates. After incubation for 3 weeks at 37 ℃, the colony morphology was recorded with the HP scanner. The H37Rv-WT was used as a control and the same treatment was performed. For scanning electron microscope analysis, the bacteria were collected by centrifugation at 500 rpm for 5 min. Then 2.5% glutaraldehyde was added into the pellet for 24 h for fixation purpose. Followed by treatment with 1% osmium tetroxide and ethanol gradient, the samples were sprayed with gold film and observed in a SU8010 scanning electron microscope (Hitachi, Japan) [12].

### High performance liquid chromatography (HPLC)

The concentration of threonine in the growth medium was quantified with High Performance Liquid Chromatography. Briefly, the freshly cultured mycobacteria, (16 days for *Mtb* and 4 days for Msm), were centrifuged at 12,000*g* for 15 min, and the supernatant were obtained and filtered with a disposable 0.22 μm cellulose acetate. After derivatization, the mixture was transferred into a 100 µL glass insert in an amber vial and analyzed by HPLC (Rigol L3000-system, Rigol, Beijing, China) as described by Priscila del Campo et al. [13]. Norleucine was used as an internal standard for assessing the recovery of amino acid from liquid medium.

### Survival of Rv3737 knockout strain and Rv3737-overexpressing strain in RAW 264.7

H37RvΔRv3737 and H37Rv-WT were infected to 5 × 10^5^/well RAW 264.7 cells in 6-well plate at multiplicity of infection (MOI) 10. After 4 h of incubation, all extracellular bacteria were removed gently by washing and intracellular bacteria were harvested. At 24 and 72 h after infection, both extracellular bacteria released from macrophage lysed in supernatant and intracellular bacteria in intact cell layer were harvested. Bacteria at 4 h, 24 and 72 h were plated on 7H10 agar plates in triplicate, plates were incubated for 3 weeks at 37 °C and CFU (colony forming unit) were counted [14].

Msm/pMV261-Rv3737 and Msm/pMV261 were infected to 5 × 10^5^/well RAW 264.7 cells in 6-well plate at multiplicity of infection (MOI) 10. After 4 h of incubation, all extracellular bacteria were removed gently by washing and intracellular bacteria were harvested. At 0 h, 6 h, 12 h, 24 and 48 h after infection, both extracellular bacteria released from macrophage lysed in supernatant and intracellular bacteria in intact cell layer were harvested. Bacteria at 0 h, 6 h, 12 h, 24 and 48 h were plated on 7H10 agar plates in triplicate, plates were incubated for one week at 37 °C and CFU (colony forming unit) were counted.

### Cytokine measurement

Culture supernatants and sediments from *Mtb*-infected RAW 264.7 cells were harvested at 0 h, 4 h, 8 h, 12 h, 24 h post-infection and stored at − 80 °C for cytokine measurement. The concentrations of TNF-α and IL-6 in culture supernatant were detected using an enzyme-linked immunosorbent assay (ELISA) kit according to the manufacturer’s instructions (Solarbio, Beijing, China) [15]. The mRNA level of TNF-α and IL-6 in culture sediments were determined using qPCR. In simple terms, the total RNA was extracted with Trizol method according to the instructions of the manufacturers [16]. After treatment with DNaseI (TaKaRa, Dalian, China), the cDNAs were reverse-transcribed from 5 µg of total RNA with the PrimeScript™ II 1st Strand cDNA Synthesis Kit (TaKaRa, Dalian, China). Real time PCR (qPCR) was carried out in triplicates for each sample using TB Green^®^ Premix Ex Taq™ II (TaKaRa, Dalian, China) in the CFX96 touch Real-Time PCR System (Bio-Rad) [17]. Primers for transcriptional level analysis are listed in Additional file 3: Table S2. GAPDH were used as the internal control of the respective qPCR experiments.

### Rv3737 expression of clinical isolates and H37Rv-WT

The *Mtb* isolates at log phase were lysed by ultrasound and then subjected to RNA extraction. The mRNA level of *Rv3737* in clinical isolates and H37Rv-WT was determined using qPCR as method above. Primers for transcriptional level analysis are listed in Additional file 3: Table S2 and SigA were used as the internal control of the respective qPCR experiments.

### Sputum collection, bacterial growth and bacterial load measurement in sputum

Sputum specimens were collected for acid-fast staining and GeneXpert MTB/RIF assay (Cepheid, Sunnyvale, CA, USA). Acid-fast staining microscopy was performed directly on all samples as described previously [18]. One milliliter sputum was mixed with 2 ml sample reagent, and incubated at room temperature for 15 min. Then the decontaminated sample was then added to a test cartridge and loaded onto the Xpert instrument. Results were reported as the cycle threshold (Ct) values that represented the minimal PCR cycles required for detection threshold [19]. The average Ct values of five probes were used to estimate bacterial load after exclusion of any delayed values due to rifampicin resistance [20]. 1.0 ml of sputum specimen with positive results from both tests were treated with *N*-acetyl-L-cysteine-NaOH-Na citrate (2.00% final concentration). After neutralization and centrifugation, the suspension of the pellet was inoculated on Lowenstein-Jensen (L-J) medium. The visible growth of colonies on L-J medium was identified using conventional biochemical method [14].

### Statistical and analysis

GraphPad Prism v7.03 (GraphPad Software, San Diego, CA, USA) was used to analyze the data and generate graphs. *t* test was utilized to compare two groups of data, two-way ANOVA was used for three or more groups of data, considering *p* value < 0.05 to be significant. The linear relationships were analysed by the R squared correlation method. Spearman Coefficient was conducted to establish the relationship between the expression level of *Rv3737* and the Ct value yielded by Xpert and between the expression level of *Rv3737* and the number of cavities.

## Results

### ***Rv3737*** affects the growth rate and morphology of ***Mtb***

.

**Fig. 2:**
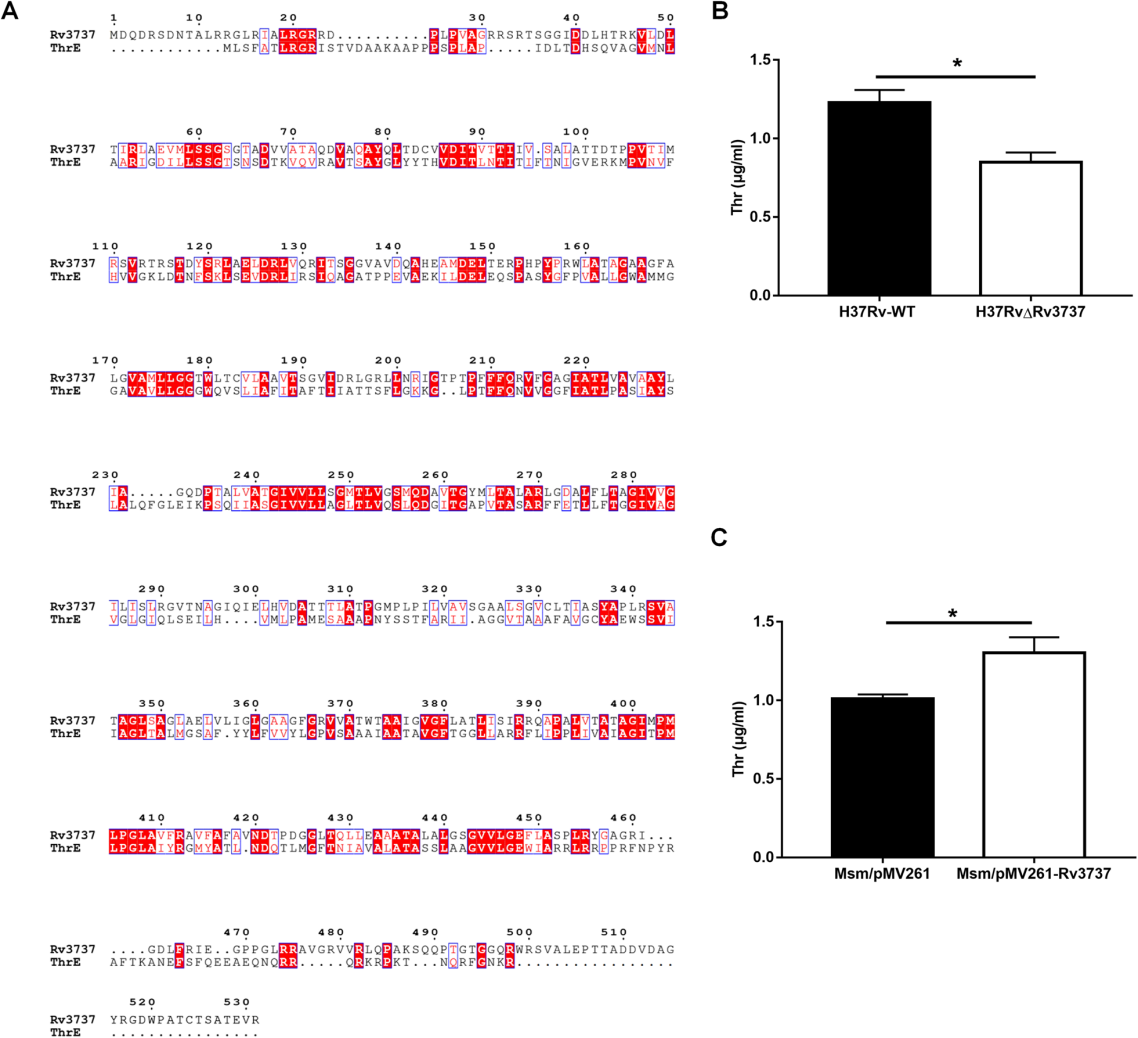
Sequence alignment of ***Rv3737*** sequences in ***Mtb*** and Determination of free threonine concentration. **A** Searched the protein database at the National Center for Biotechnology Information for Rv3737 in *Mtb* and ThrE in *C. glutamicum* amino acid sequences. ClustalW software (http://www.clustal.org/) was used to align sequences and reconstruct a phylogenetic tree based on sequence similarities. Shared amino acids are highlighted in red selected for testing in this study. **B** The free threonine concentration of H37Rv-WT and H37RvΔRv3737 in 7H9 medium grown for 16 days. **C** The free threonine concentration of Msm/pMV261 and Msm/pMV261-Rv3737 in 7H9 medium grown for 4 days. The difference between the wild type and the mutant was significant by Student’s *t* test (*p < 0.05; **p < 0.01; ***p < 0.001)

**Fig. 3:**
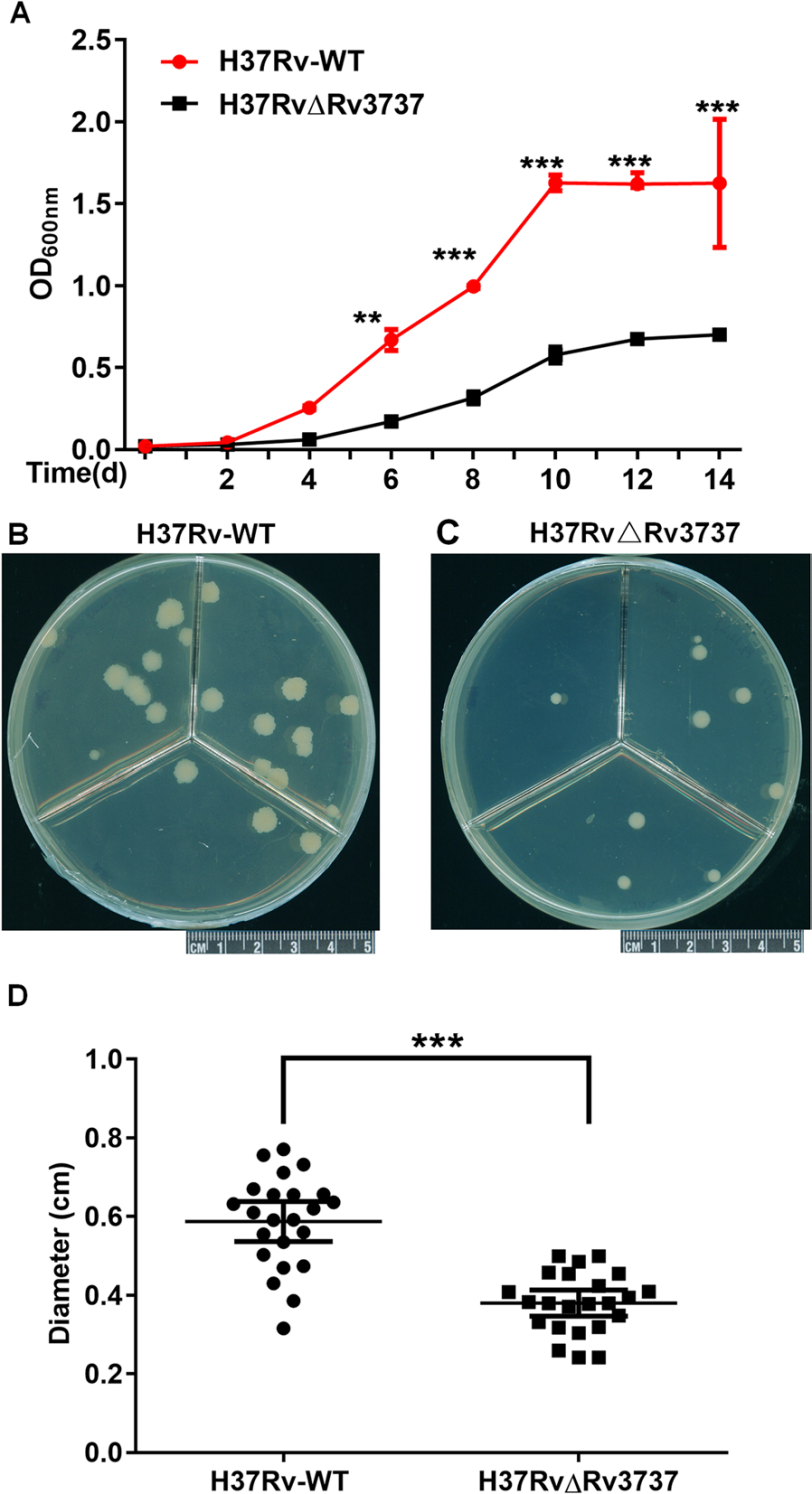
In vitro growth of H37RvΔRv3737. **A** Growth curve of H37Rv-WT and H37RvΔRv3737 in 7H9 medium. **B** Colonies of H37Rv-WT and H37RvΔRv3737 on 7H10 agar grown for 21 days. **C** Diameter of colonies of H37Rv-WT and H37RvΔRv3737 on 7H10 agar. Experiments were performed in triplicates. The difference between the wild type and the mutant was significant by Student’s *t* test (*p < 0.05; **p < 0.01; ***p < 0.001)

At the amino acid level, *Rv3737* shared 29.60% sequence identity with ThrE of *C. glutamicum* (Fig. 2A). We have constructed H37RvΔRv3737 and Msm/pMV261-Rv3737 at the same time. The concentration of threonine in culture medium were compared across different strains (Fig. 2B, C). Our data revealed that the free threonine concentration of the culture medium where the H37RvΔRv3737 (0.85 ± 0.06 µg/ml) grew was remarkedly decreased compared to that of H37Rv-WT strain (1.23 ± 0.08 µg/ml, *p < *0.05), while the overexpression of Rv3737 resulted in a significant elevation of threonine concentration in *M. smegmatis* (1.30 ± 0.10 µg/ml for Msm/pMV261-Rv3737 verses 1.01 ± 0.03 µg/ml for Msm/pMV261, *p <* 0.05). We then assessed whether inactivation of *Rv3737* affects the in vitro growth and physiology of *Mtb*. As shown in Fig. 3A, the H37RvΔRv3737 strain exhibited significantly slow growth rate compared to H37Rv-WT strain in standard culture medium. Colony size of H37RvΔRv3737 and H37Rv-WT strain was compared by plating the same dilution on plates after 21 days (Fig. 3B, C). The average colony size of H37RvΔRv3737 was 0.38 ± 0.02 μm, which was much smaller than that of H37Rv-WT strain (0.58 ± 0.02 μm, Fig. 3D).

**Fig. 4:**
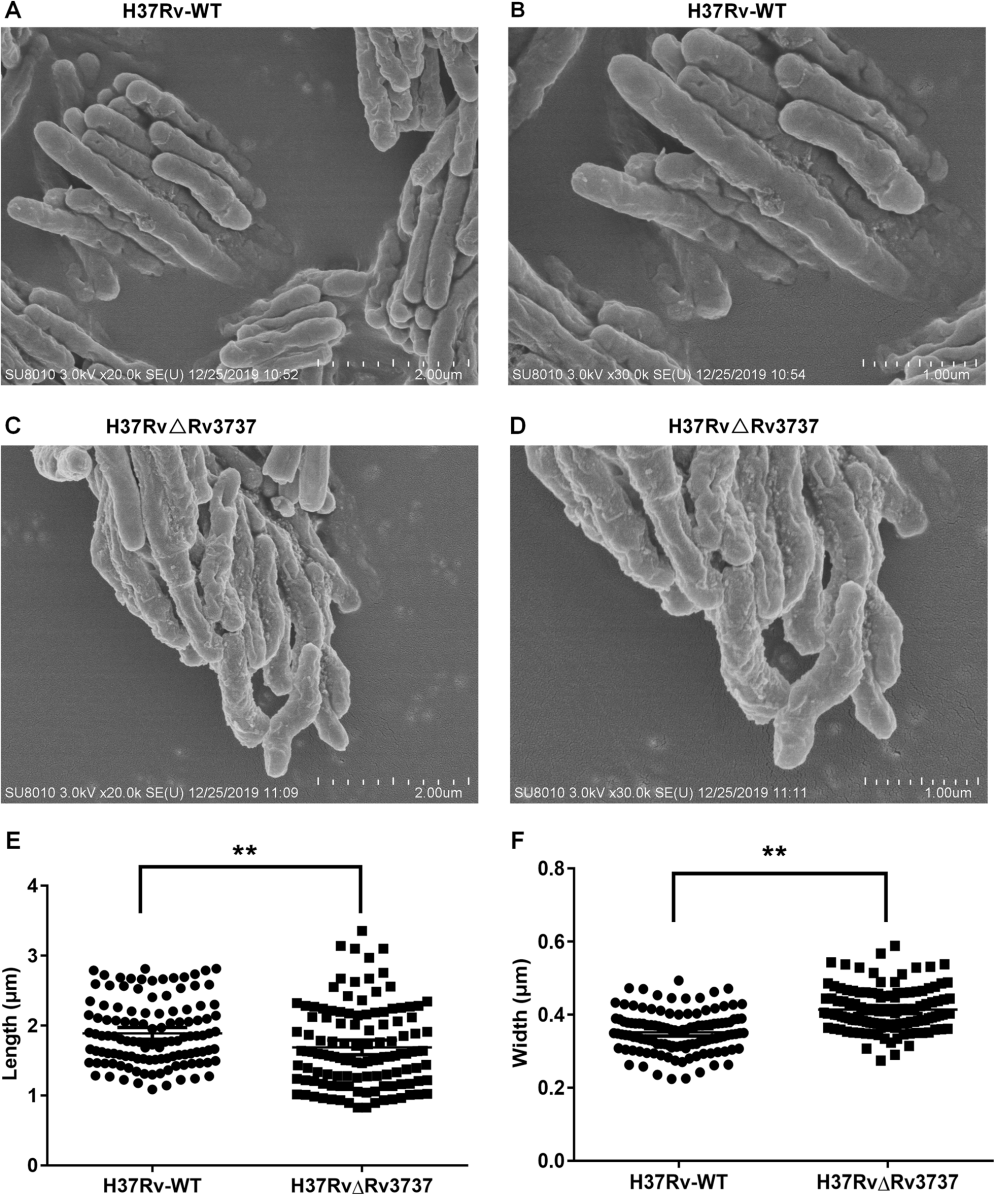
Morphological changes between the H37Rv-WT and H37RvΔRv3737 strains. ** A**–**D** Scanning electron microscope photographs of strains. Bacteria were grown in 7H9 medium. **E** Length of H37Rv-WT and H37RvΔRv3737. **F** Width of H37Rv-WT and H37RvΔRv3737. The difference between the wild type and the mutant was significant by Student’s *t* test (*p < 0.05; **p < 0.01; ***p < 0.001)

Field emission scanning electron microscopy was conducted to assess cell morphology of *Mtb*, and measured the length and width of the cell with Image J, each strain has no less than 120 bacteria. Individual cells from two strains were indistinguishable in morphology characteristics, whereas significant differences were observed in cell lengths. As noted in Fig. 4, the average cell length of the H37Rv-WT and H37RvΔRv3737 were 1.89 ± 0.04 and 1.69 ± 0.05 μm (*p < *0.05), respectively, suggesting that the latter had a shorter cell length. In contrast, the average cell width of H37RvΔRv3737 was 0.41 ± 0.01 μm, which was statistically higher than that of H37Rv-WT strain (0.35 ± 0.01 μm, *p *< 0.05).

### ***Rv3737*** promotes the survival of ***Mtb*** in macrophages

**Fig. 5:**
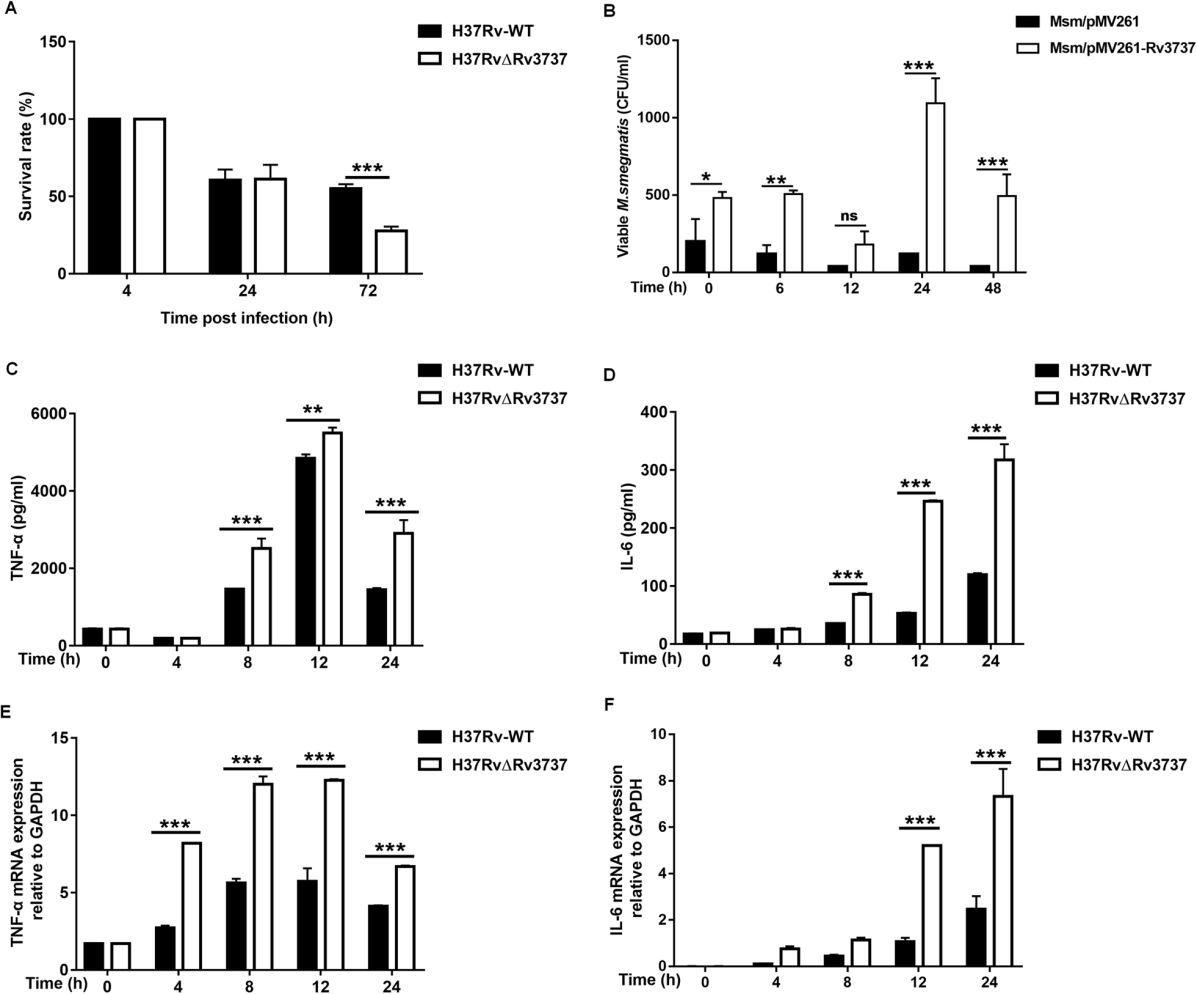
Survival of H37RvΔRv3737 and Msm/pMV261-Rv3737 in macrophages and the effects on pro-inflammatory cytokines. **A** Survival analysis of H37Rv-WT and H37RvΔRv3737 in macrophages. **B** Survival analysis of Msm/pMV261 and Msm/pMV261-Rv3737 in macrophages. **C**, **D** The mRNA levels of TNF-α and IL-6 in macrophages infected with H37Rv-WT and H37RvΔRv3737. Experiments were performed in triplicates. **E**, **F** Immunoassays for TNF-α and IL-6 in supernatants collected from macrophages infected with H37Rv-WT and H37RvΔRv3737. The difference between the wild type and the mutant was significant by Student’s *t* test (*p < 0.05; **p < 0.01; ***p < 0.001)

Mouse macrophages were infected with H37Rv-WT, H37RvΔRv3737, Msm/pMV261, and Msm/pMV261-Rv3737 to determine differences in capacity for intracellular growth. As shown in Fig. 5A, B, the intracellular survival was assessed at 24 and 72 h after infection at 4 h as reference. The survival rate of H37Rv-WT strain was 2 folds higher than that of H37RvΔRv3737 at 72 h, respectively. However, the survival of Msm/pMV261-Rv3737 in macrophages was significantly increased relative to that of Msm/pMV261. Taken together, these data indicated that *Rv3737* had an important role in enhancing the infection capability and intracellular survival of tubercle bacilli.

### Rv3737 inhibits the release of TNF-α and IL-6 in macrophages

To explore the potential role of *Rv3737* in modulating the innate immune response, we investigated the levels of cytokines upon infection of RAW264.7 cells with H37Rv-WT and H37RvΔRv3737. A significantly higher level of TNF-α and IL-6 mRNA expression was observed in macrophages infected with H37RvΔRv3737 as compared to H37Rv-WT (Fig. 5C, D). Detection of cytokines in the culture supernatants of macrophages also supported the elevated secretion of proinflammatory cytokines (TNF-α and IL-6) at increasing time points (Fig. 5E, F).

### Correlation between ***Rv3737*** expression and disease severity

**Fig. 6:**
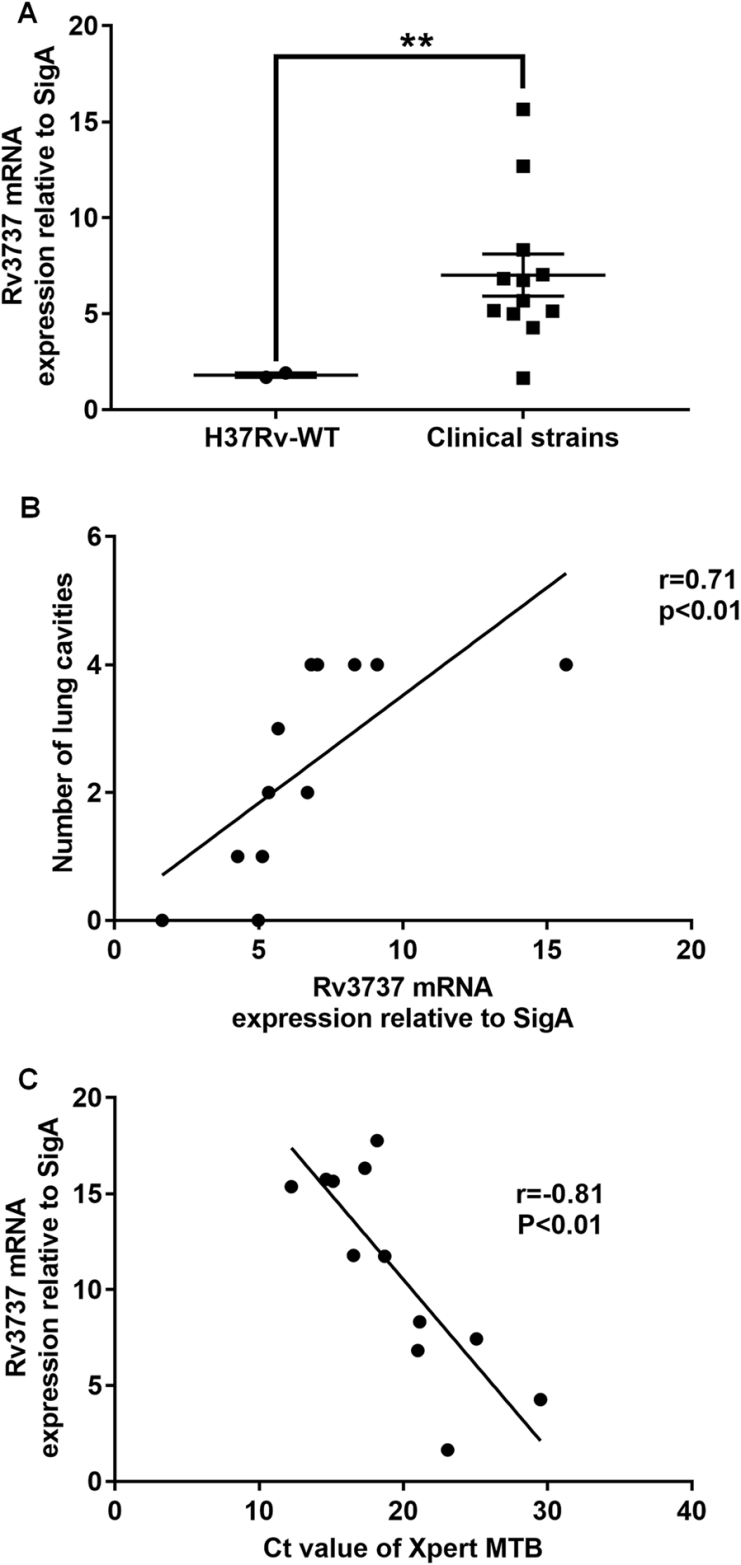
Correlation between Rv3737 mRNA level and disease severity. **A** Comparison of *Rv3737* mRNA levels between H37Rv-WT and clinical isolates. **B** Relationship between the expression level of *Rv3737* and the C(t) value yielded by Xpert. **C** Relationship between the expression level of *Rv3737* and the number of cavities. Each assay was performed in triplicate. The difference between the wild type and the mutant was significant by Student’s *t* test (*p < 0.05; **p < 0.01; ***p < 0.001). The relationship between the expression level of *Rv3737* and the C(t) value yielded by Xpert and between the expression level of *Rv3737* and the number of cavities was established by Spearman Coefficient (*p* < 0.01)

Based on the slower in vivo growth and increased host proinflammatory cytokine response, we hypothesized that the expression level of *Rv3737* correlated with *Mtb* virulence in the host. In order to test this hypothesis, we recruited 12 clinical *Mtb* isolates to determine whether the upregulation of *Rv3737* could lead to more severe clinical symptoms. A total of 12 patients infected with diagnosed TB were retrospectively included in our analysis (Table 1). As shown in Fig. 6A, *Rv3737* expression was significantly increased in clinical *Mtb* isolates than H37Rv-WT. Of note, the relative expression level of *Rv3737* was positively correlated with lung cavity number of TB patients (r = 0.71, *p* < 0.01, Fig. 6B). Similarly, the higher *Rv3737* mRNA level resulted in lower C(t) value by Xpert MTB/RIF assay, demonstrating that a positive correlation between *Rv3737* expression and bacterial load in TB patients (r = − 0.81, *p* < 0.01, Fig. 6C).

**Table 1:** Demographic and clinical characteristics of clinical cases

Characteristics	No. (%) of subjects
Total	12 (100.00)
Sex	
Male	9 (75.00)
Female	3 (25.00)
Age, years	
> 6	4 (33.33)
> 34	6 (50.00)
> 64	2 (16.67)
BMI, kg/m^**2**^	
< 18.5	9 (75.00)
18.5–23.9	3 (25.00)
> 23.9	0 (0.00)
HIV	0 (0.00)
Symptom	
Fever	1 (8.33)
Night sweat	2 (16.67)
Fatigue	3 (25.00)
Cough	10 (83.33)
Haemoptysis	0 (0.00)
Chest pain	4 (33.33)
Severe dyspnoea	2 (16.67)
Treatment history	
New case	5 (41.67)
Previously treated case	7 (58.33)
Complications	
COPD	2 (16.67)
HBV	1 (8.33)
Heart disease	1 (8.33)
Xpert MTB/RIF	
MTB + / RIF −	7 (58.33)
MTB + / RIF +	5 (41.67)
Number of cavities (chest CT)	
0	2 (16.67)
1	1 (8.33)
2	2 (16.67)
3	3 (25.00)
≤ 4	4 (33.33)
Genotypic	
Beijing	6 (50.00)
Non-Beijing	6 (50.00)

## Discussion

Transporter systems are commonly considered as a potential tool for delivery of therapeutic agents. Recently experimental studies reveal that several transporters be required for chronic infection and expression of virulence in pathogenic bacteria [21, 22]. In this study, we attempted to understand the possible role of transporter protein Rv3737, which alters in vitro growth and intracellular survival of bacteria inside macrophages. Depletion of Rv3737 in *Mtb* resulted in decreased growth rate in vitro compared to H37Rv-WT; however, the mutant strain displayed usual rough and dry colonies as control strain, indicating that Rv3737 might be not involved in the cell wall lipid remodeling in *Mtb*. Although the exact characteristics and role of this transporter in growth profile remain unclear, we speculate that the inactivation of Rv3737 might lead to accumulation of metabolic waste products in vivo and consequently inhibit their growth.

Experimental evidence from previous studies confirms that highly virulent *Mtb* isolates have faster in vivo doubling time [23, 24]. We found that the knockout of *Rv3737* had a markedly negative impact on intracellular survival as compared to the control. On one hand, this fact may reflect the declined growth rate of tubercle bacilli in macrophages, as demonstrated in vitro observations. On the other hand, the elevated levels of proinflammatory cytokines were noted in the culture supernatant of macrophages infected with H37RvΔRv3737, thus promoting intracellular bacteria clearance in macrophages. Specific mechanism behind this significant correlation is presently unclear. On the basis of its putative transporter function, the molecules exported by Rv3737 into extracellular substance were able to impair host defense against intracellular bacteria via inhibiting inflammatory response. On the basis of our findings, Rv3737 may participate in modulation of reduced or delayed host proinflammatory cytokine response, which is required for persisting virulence and survival of *Mtb* within host macrophages.

Furthermore, the elevated expression level of *Rv3737* was noted in clinical *Mtb* isolates as compared to H37Rv-WT with attenuated virulence. This diversity supports our previous findings that *Rv3737* may be involved in virulence of *Mtb*. Notably, a significant positive correlation between *Rv3737* expression level and bacterial load in pulmonary TB patients raises the possibility that the isolates with increased expression of *Rv3737* are prone to escape clearance by alveolar macrophages, thus leading to greater bacillary multiplication in the host. The high bacterial burden always causes more lung damage and higher mortality [25, 26]. In line with previous observation, we observed that the higher expression of *Rv3737* was most likely to result in more cavities among patients affected by pulmonary TB. In view of the strong association between *Rv3737* and lung pathology, we speculate that is could be used as a candidate biomarker for predicting the virulence of distinct isolates, and its potential pathogenic effect in host.

We also acknowledged several obvious limitations to the present study. First, we observed the slower growth rate and shorter length of H37RvΔRv3737; whereas the mutant had larger width than H37Rv-WT control, which may be associated with the remodeling of cytoskeleton to modulate the bacterial growth. However, the reason for this phenomenon remains unclear. Second, despite validating the potential role of Rv3737 as transporter of threonine in *Mtb*, we were unable to successfully construct the *Mtb* strains with overexpression of Rv3737, indicating the expression balance of this gene was subtly regulated in tubercle bacilli. As an alternative, the Rv3737-overexpressed M. smegmatis model was used to testify its function for mycobacteria. Third, in vitro findings were not verified with animal model, which weakened the significance of conclusion. Fourth, the pathological pattern of pulmonary TB is a complicated process between host and pathogen, and multiple factors may bias our conclusion, such as genetic lineage of *Mtb*, immunology status of host and sample size of validation cohort. Thus the primary correlation between the expression of Rv3737 and pathological patterns is required to confirm in the future. Finally, although we recruited active TB cases in our analysis, the diverse courses of TB disease across patients may serve as a major challenge that biases our conclusion.

## Conclusions

In conclusion, our data firstly demonstrate that the threonine transporter Rv3737 is required for in vitro growth and survival of bacteria inside macrophages. In addition, the expression level of *Rv3737* may be associated with bacterial load and disease severity in pulmonary tuberculosis patients. Further studies will be conducted to elucidate the molecular mechanism of this transporter to *Mtb* virulence.

## Supplementary Information

**Additional file 1:** Full length gels and blots image with changes marked of Fig. 1D.

**Additional file 2:** **Table S1.** Bacterial strains, plasmids and cells used in this study.

**Additional file 3:** **Table S2.** List of primer used in this study.

## Data Availability

The datasets used and/or analysed during the current study are available from the corresponding author on reasonable request.

## Abbreviations

ANOVA: Analysis of variance
CFU: Colony forming units
cDNA: Complementary deoxyribonucleic acid
DMEM: Dulbecco’s modified eagle medium
ELISA: Enzyme-linked immunosorbent assay
GAPDH: Glyceraldehyde-3-phosphate dehydrogenase
HIV: Human immunodeficiency virus
IL: Interleukin
LB: Luria-Bertani
L-J: Lowenstein-Jensen
Mtb /MTB: *Mycobacterium tuberculosis*
MOI: Multiplicity of infection
mRNA: Messenger ribonucleic acid
OD: Optical density
OADC: Oleic albumin dextrose catalase
PCR: Polymerase chain reaction
qPCR: The real-time reverse transcription polymerase chain reaction
RIF: Rifampin
TNF-α: Tumor necrosis factor-α
TB: Tuberculosis
WT: Wide type
