# Supplementary figures and images for: Rv3737 is required for Mycobacterium tuberculosis growth in vitro and in vivo and correlates with bacterial load and disease severity in human tuberculosis
Source: BMC Infect Dis. 2022 Mar 14;22:256. doi: 10.1186/s12879-021-06967-y (PMC8919692; doi:10.1186/s12879-021-06967-y)

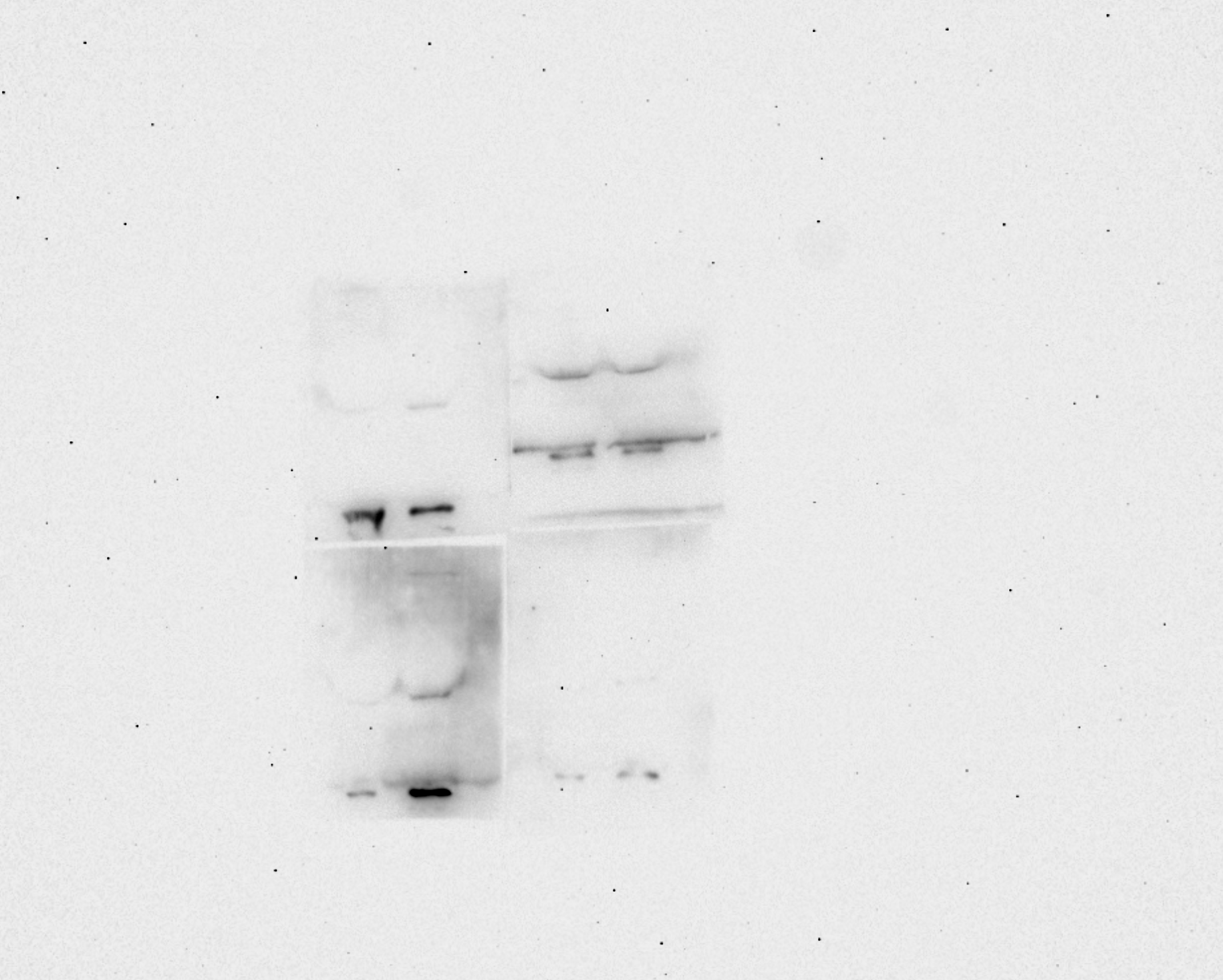

Supplement: Supplementary file 1 — Additional file 1: Full length gels and blots image with changes marked of Fig. 1D. [file 12879_2021_6967_MOESM1_ESM.tif]
