# Supplementary material for: Rv3737 is required for Mycobacterium tuberculosis growth in vitro and in vivo and correlates with bacterial load and disease severity in human tuberculosis
Source: BMC Infect Dis. 2022 Mar 14;22:256. doi: 10.1186/s12879-021-06967-y (PMC8919692; doi:10.1186/s12879-021-06967-y)
Supplement: Supplementary file 2 — Additional file 2: Table S1. Bacterial strains, plasmids and cells used in this study. [file 12879_2021_6967_MOESM2_ESM.docx]

**Table S1. Bacterial strains, plasmids and cells used in this study**

| **Strains/plasmids/cells** | **Description** | **source/reference** |
| --- | --- | --- |
| **Strains** |  |  |
| Wild-type *Mtb* H37Rv | Pathogenic; for amplifying *M. tuberculosis* Rv3737 gene | Guizhou provincial Center For Disease Control And Prevention |
| *M.smegmatis* mc^2^  155 | Nonpathogenic; for amplifying *M. tuberculosis* Rv3737 gene and achieving homologous recombination at *rmlA* locus | Wuhan Institute of Virology, CAS |
| *E. coli* DH5α | For constructing plasmids | Tsingke biological tachnology |
| *E. coli* HB101 | For constructing plasmids | Tsingke biological tachnology |
| H37RvΔRv3737 | *M. tuberculosis* Rv3737 knockout strain | This work |
| Msm/pMV261 | pMV261 empty plasmid strain | This work |
| Msm/pMV261-Rv3737 | Rv3737-overexpressing strain | This work |
| *Clinical Mtb* isolates | Collected from a cohort of participants with pulmonary TB | Affiliated Hospital of Zunyi Medical University |
| **Plasmids** |  |  |
| p0004s | For constructing plasmids | Wuhan Institute of Virology, CAS |
| phAE159 | For constructing plasmids | Wuhan Institute of Virology, CAS |
| p0004s-LR | For constructing plasmids | This work |
| phAE159-p0004s-LR | For constructing plasmids | This work |
| pMV261 | For overexpression | Affiliated Hospital of Zunyi Medical University |
| **Cells** |  |  |
| RAW264.7 | Mouse mononuclear macrophage leukemia cells | American Type Culture Collection |
