# Supplementary material for: Rv3737 is required for Mycobacterium tuberculosis growth in vitro and in vivo and correlates with bacterial load and disease severity in human tuberculosis
Source: BMC Infect Dis. 2022 Mar 14;22:256. doi: 10.1186/s12879-021-06967-y (PMC8919692; doi:10.1186/s12879-021-06967-y)
Supplement: Supplementary file 3 — Additional file 3: Table S2. List of primer used in this study. [file 12879_2021_6967_MOESM3_ESM.docx]

**Table S2. List of primer used in this study**

| S1.No | Name of primer | Sequence(5’-3’) | Product length |
| --- | --- | --- | --- |
| 1^a^ | LFP | TTTTTTTTGGCCTAAATGGCCCCCAGTTATCGCCATCCG | 781 |
| 2^a^ | LRP | TTTTTTTTGGCCTTTCTGGCCCAATGCCGTGTTGTCCGA |  |
| 3^a^ | RFP | TTTTTTTTGGCCTAGATGGCCCCAAGAGCCAGCAGCCGA | 640 |
| 4^a^ | RRP | TTTTTTTTGGCCTCTTTGGCCGTCTTTGCGGTCCTGGCG |  |
| 5^b^ | LYZFP | CAATGGATTTCGGGAGAC | 1064 |
| 6^b^ | LYZRP | GTGGACCTCGACGACCCTAG |  |
| 7^b^ | RYZFP | TGGATCTCTCCGGCTTCACC | 1064 |
| 8^b^ | RYZRP | TACTGTTCAACGGCGGTG |  |
| 9^c^ | Rv3737 For | GTTCGCCGTCAATGACACAC | 115 |
| 10^c^ | Rv3737 Rev | CAATGGTGAGGCGAGGAACT |  |
| 11^c^ | M.tb-sigA For | CTCGGTTCGCGCCTACCTCA | 130 |
| 12^c^ | M.tb-sigA Rev | GCGCTCGCTAAGCTCGGTCA |  |
| 13^d^ | TNF-α For | AAGCCTGTAGCCCACGTCGTA | 122 |
| 14^d^ | TNF-α Rev | GGCACCACTAGTTGGTTGTCTTTG |  |
| 15^d^ | IL-6 For | ACAACCACGGCCTTCCCTACTT | 129 |
| 16^d^ | IL-6 Rev | CACGATTTCCCAGAGAACATGTG |  |
| 17^d^ | GAPDH For | GAGCCAAACGGGTCATCATCT | 232 |
| 18^d^ | GAPDH Rev | GAGGGGCCATCCACAGTCTT |  |
| 19^e^ | pMV-Rv3737-ECOR I-For | CCGGAATTCCGGATGGACTACAAAGACGATGACGACAAG GATCAAGATCGATCGGACAACAC | 1590 |
| 20^e^ | pMV-Rv3737-Hind III-Rev | CCCAAGCTTGGG CTAGCGCACCTCGGTCGCG |  |

Note：^a^ Primers are used in PCR to construct the left and right arms of the target gene

^b^ Primers are used in PCR to verify the successful construction of the knockout strain

^c^ Primers are used in qPCR to verify the successful construction of the knockout strain

^d^ Primers are used in qPCR to detect inflammatory factor expression

^e^ Primers are used in PCR to amplify the entire gene sequence of Rv3737 and used for overexpression
